# Perceptions and Knowledge towards COVID-19 Vaccine Hesitancy among a Subpopulation of Adults in Kenya: An English Survey at Six Healthcare Facilities

**DOI:** 10.3390/vaccines10050705

**Published:** 2022-04-29

**Authors:** Jasmit Shah, Abdulaziz Abeid, Karishma Sharma, Soraiya Manji, Jamila Nambafu, Robert Korom, Keya Patel, Mohamed Said, Mohamed Ali Mohamed, Mohamed Sood, Victor Karani, Patrick Kamandi, Sarah Kiptinness, Ryan T. Rego, Rajiv Patel, Reena Shah, Zohray Talib, Sayed K. Ali

**Affiliations:** 1 Department of Medicine, Aga Khan University, Nairobi P.O. Box 30270-00100, Kenya; jasmit.shah@aku.edu (J.S.); keyadp@icloud.com (K.P.); rajiv.patel@aku.edu (R.P.); reena.shah@aku.edu (R.S.); 2 Brain and Mind Institute, Nairobi Campus, Aga Khan University, Nairobi P.O. Box 30270-00100, Kenya; 3 Department of Medicine, Coast General Teaching and Referral Hospital, Mombasa P.O. Box 90231-80100, Kenya; azyzabeid@gmail.com (A.A.); mohammad.dhiyebi@gmail.com (M.A.M.); msood1@hotmail.com (M.S.); 4 Department of Medicine, Avenue Hospital, Nairobi P.O. Box 45280-00100, Kenya; karishmasharma313@gmail.com (K.S.); mohas.ke@gmail.com (M.S.); 5 Clinical Research Unit, Department of Hematology and Oncology, Aga Khan University Hospital, Nairobi P.O. Box 30270-00100, Kenya; 6 Department of Medicine, Mediheal Hospital Parklands, Nairobi P.O. Box 39698-00623, Kenya; soraiyamanji@gmail.com; 7 Department of Medicine, PCEA Chogoria Hospital, Tharaka-Nithi P.O. Box 35-60401, Kenya; njamilaw@gmail.com (J.N.); victorkanake77@gmail.com (V.K.); patrohmumo@gmail.com (P.K.); 8 Penda Health Ltd., Nairobi P.O. Box 22647-00100, Kenya; robert@pendahealth.com (R.K.); sarah.kiptinness@pendahealth.com (S.K.); 9 Center for Global Health Equity, University of Michigan, Ann Arbor, MI 48105, USA; regor@med.umich.edu; 10 Department of Medical Education, California University of Science and Medicine, Colton, CA 92324, USA; zmtalib@gmail.com

**Keywords:** COVID-19, vaccine hesitancy, Kenya

## Abstract

Background: Vaccine hesitancy, as defined by the WHO, is the reluctance or refusal to vaccinate despite the availability of vaccines and is one of the ten threats to global health in 2019. Vaccine hesitancy remains a complex matter influenced by multiple factors, especially in sub-Saharan Africa. Methods: We conducted a cross-sectional study between November 2021 and January 2022 among the general adult public seeking care at six different healthcare facilities in Kenya. The survey, in English, consisted of questions based on demographics, knowledge, and attitudes, including hesitancy towards the COVID-19 vaccine. Results: Of the 3996 surveys collected, 55.1% were from private, 19.5% from faith-based and 25.3% from government facilities., Approximately 81.0% of all the participants reported it was important to get a vaccine to protect other people from COVID-19, 79.9% reported they would take a vaccine to protect against COVID-19, yet 40.5% reported being hesitant to take the vaccine primarily due to side effects. Most of the variables were associated with receiving a vaccine. Only 52.1% of those seeking care from the government facility and 54.5% of those seeking care from the faith-based facility were vaccinated, compared to 81.5% seeking care from the private facilities (*p* < 0.001). More participants from private facilities felt that vaccines are safe as compared to those at the faith-based and government facilities (*p* < 0.001). Conclusion: Vaccine hesitancy in Kenya, even though much lower than reported in other countries, remains a dynamic problem. Mitigating strategies specific to Africa need to be developed to help address vaccine hesitancy in this part of the continent.

## 1. Introduction

As of 2021, more than 8 million confirmed cases of COVID-19 were reported within the African continent, with more than 150,000 deaths, of which more than 320,000 cases and about 5500 deaths were in Kenya [1]. One of the greatest tools in combating COVID-19 morbidity and mortality is the COVID-19 vaccine. As of 2021, 9.17 billion doses of the COVID-19 vaccine had been administered worldwide, with 3.82 billion people fully vaccinated. Vaccination coverage is varied widely by income group, with average coverage of 58.49 doses/100 people, 17.30 doses/100 people, 11.95 doses/100 people, and 1.26 doses/100 people for high, upper middle, lower middle, and low income countries, respectively [2]. In Kenya specifically (as at February 2022), 16.6 million doses had been given and 7.58 million (14.1%) of the total population had been fully vaccinated. According to the United Nations website UN News: Global Perspective Human Stories, 832 million vaccine doses had been administered by April 2021 and 82% had gone to high and upper middle income countries, while only 0.2% had been delivered to low and middle income countries (LMICs) [3]. This inequality of healthcare resources, especially during a pandemic, certainly adds to the mistrust in healthcare systems and could promote vaccine hesitancy among people in LMICs [4]. Vaccine inequity remains a challenging issue around the world, especially in LMICs. Given the limited supply, vaccine hesitancy becomes a critical issue to mitigate to ensure the optimal uptake in low resource settings.

Various factors contribute to inequality in vaccine distribution, chief among which is supply and distribution of vaccines. High income countries (HICs), often the manufacturers and preferred customers of pharmaceutical firms, often hoard vaccines. Further, due to high costs of vaccines, LMICs often cannot afford them. However, with vaccination rates now slowing in high income countries, the cost of vaccines falling, and actions through global mechanisms such as COVAX, LMICs are now starting to see an increase in vaccine supply. To get these vaccines finally into the arms of those who need them, low income countries now have to focus on distribution: supply chains, personnel, and what we will discuss in this paper, patient level decision-making and vaccine hesitancy.

Vaccine hesitancy, as defined by the WHO, is the reluctance or refusal to vaccinate despite the availability of vaccines and is one of the ten threats to global health in 2019. Some of the reasons why people chose not to get vaccinated include a lack of trust in the healthcare system, complacency and inconvenience in getting the vaccine [5]. A framework to better understand the five psychological antecedents of vaccination called the 5C model includes: confidence, complacency, convenience, risk calculations, and collective responsibility. This framework, developed mainly in high income countries, harnesses the potential to better understand and come up with strategies to address vaccine hesitancy, but might miss factors that could be pertinent to LMICs. Large knowledge gaps around vaccine hesitancy still remain, especially in Africa, due to limited tools, especially those that have been validated in local or regional context [6,7].

Information on social media outlets is not scientifically filtered and has the potential to misguide. Early studies have already suggested that disinformation about the vaccine posted on various social media outlets appears to have significantly contributed to vaccine hesitancy and the lack of uptake. Wilson and Wiysonge in their study looking at social medial and vaccine hesitancy found that a rise in the number of negative tweets around vaccines related to foreign disinformation campaigns [8]. A number of conspiracy theories appear to have contributed, including one that claims the use of the vaccine is aimed to disable local populations so the “West” can control resources in Africa. Another conspiracy theory is based on the misinformation that vaccines contain microchips that are implanted to control the worlds’ population [9,10,11]. Lucia et al. (2021) found that 23% of US medical students were vaccine hesitant, due to fear of side effects and mistrust of the public health system [12]. Similarly, Salali and Uysal found that 31% of the participants in Turkey and 14% in the UK were not convinced about getting the COVID-19 vaccine, due to misinformation regarding the origin of COVID-19 [13]. Indeed, a major theme that emerged in the literature was misinformation, often stemming from social media, including misinformation on the origin of the virus, infertility, and efficacy of the vaccine [11].

While a vast majority of studies on COVID-19 vaccine decision-making and hesitancy are from HICs, there are a few studies from LMICs. Solis Arce et al. examined COVID-19 acceptance among 10 LMICs in Asia, Africa, and South America as well as Russia and the United States. They found higher willingness to get the COVID-19 vaccines in the LMICs samples as compared to Russia or the United States, but while the acceptance was primarily explained by the desire for personal protection from COVID-19, side effects of the vaccine remained a major reason for hesitancy [14]. Further, Anjorin et al. (2021) conducted a survey of 34 African countries, estimating that 63% were willing to receive the COVID-19 vaccine, 79% were concerned about side effects, and 39% were worried about COVID-19 infection after receiving the vaccine [15]. Similar results were found in studies in South Africa, Ethiopia, Egypt, Nigeria, and Somalia, with major factors of hesitancy being reported [16,17,18,19,20,21].

Kenya, a low–middle income country in sub-Saharan Africa (SSA), has a population of more than 50 million people, with 75% of this population living in rural areas and about 46% living below the poverty line. Kenya’s health budget for 2016–2017 was approximately KES 60.3 billion (4% of the total budget or 5.2% as a share of GDP, equaling USD $530 million). The majority of the healthcare spending goes towards curative therapy and only about 16% is spent on preventive actions such as vaccination, HIV/TB prevention, etc. [22,23]. There are several components to the Kenyan healthcare system; government, private, and faith-based. About half of the healthcare is provided in the public system in government-run facilities, whereas the other half is made up of private facilities, of which about 20% are faith-based organizations [24]. The Ministry of Health in Kenya is the primary supplier of vaccines. Initial policies related to vaccine distribution were based on the WHO Strategic Advisory Group of Experts (SAGE) Roadmap allowing healthcare workers who were at greater risk of infection and front-line workers to get access to the vaccine first [25]. The AstraZeneca vaccine was the first vaccine made available in Kenya in early 2021 and other approved vaccines reached the country later in 2021. Since that time, the vaccine supply has grown due to COVAX and doses are available to all Kenyans over the age of 15.

However, data on vaccine hesitancy in Africa is aging; at the time of writing, the most recent Kenyan study had taken place in August 2021 [26]. Given the rapidly changing landscape, updates of this information are needed. We conducted this study to better understand the reasons for vaccine hesitancy among the general population being treated at six different healthcare facilities in Kenya by government, private, and faith-based hospitals. We hope that this study will help inform vaccination promotion programs in Kenya and similar countries, as the COVID-19 vaccine supply increases.

## 2. Methodology

We carried out a cross-sectional study between November 2021 and January 2022. The general adult public (patients and relatives) visiting the inpatient and outpatient clinics were recruited from six different healthcare facilities: Aga Khan University Hospital, Nairobi (AKUHN), Penda Health (Nairobi), Avenue Hospital (Nairobi), Mediheal Hospital (Nairobi), Coast General Teaching and Referral Hospital (CGTRH) (Mombasa), and PCEA Chogoria Mission Hospital (Tharakanithi). Aga Khan University Hospital, Penda Health, Avenue Hospital, and Mediheal Hospital are all private hospitals. CGTRH is a government hospital, while PCEA Hospital is a not-for-profit faith-based organization.

The survey was in English and was strategically broken down into various sections including: demographics, medical history knowledge attitudes, and perspectives towards the COVID-19 pandemic and vaccination (Supplementary Materials). The survey used in this study was developed based on a literature review, questions used in other similar studies, and discussion with the research team of this study [27,28]. The survey was designed to reduce survey fatigue. To reduce potential bias of self-reported data, confidentiality of participants and privacy of their responses was prioritized. Data were collected both through online and paper-based surveys. Each site had an investigator that was responsible for administration of the survey. The site investigator, based on the resources available at the respective sites, decided to choose using either or both paper or online surveys. Each site investigator received preliminary training on administration of the survey. The survey was self-administered with the support of the site investigator. Online survey data were collected through the Research Electronic Data Capture (REDCap) platform (Vanderbilt and National Institute of Health) [29]. Online and written consent was obtained from all the participants. Approval for this study was obtained from the Institutional Ethics and Review Committee at the Aga Khan University, Nairobi, and the hospital leaderships at Penda Health, Avenue Hospital, Mediheal Hospital, Coast General Teaching and Referral Hospital, and PCEA Chogoria Mission Hospital. Participants were allowed to withdraw from the study at any time without any consequences.

Sociodemographic characteristics collected were age, gender, marital status, education, employment status, citizenship, and race. Additionally, participants were asked about their medical history and any comorbidities. COVID-19 related questions included if participants were infected with COVID-19 or knew anyone who was infected, if they had contracted the virus with or without a test and concerns or worries about the COVID-19 pandemic. The section on attitudes and perspectives towards COVID-19 vaccines consisted of questions about hesitancy and concerns, scored using a Likert scale (strongly agree, agree, neutral, disagree, and strongly disagree). The details of the full set of questions are in the Supplementary Materials.

Continuous data were analyzed using medians and interquartile ranges (IQRs) whereas categorical data were analyzed as frequencies and percentages. The non-parametric Kruskal–Wallis test was used to compare the continuous variables and Fisher’s exact test was used to compare the categorical variables between group associations. Logistic regression was also employed to identify associations and odds ratio and 95% CI was presented. Data analysis was performed using SPSS statistical software V. 20.0 (IBM, Armonk, NY, USA). The significance level was set at α = 0.05, and all tests were two-tailed.

## 3. Results

### 3.1. Demographics

**Figure 1:**
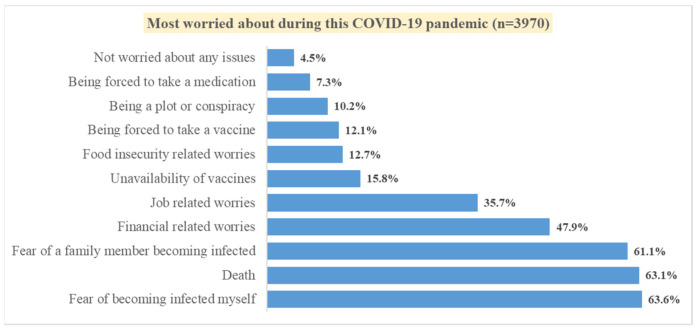
Proportions of the issues respondents worried about most during the COVID-19 pandemic.

A total of 3996 surveys were completed and included in the analysis. The median age of participants was 33.0 years (IQR: 26.5, 43.0) with 55.8% being females. More than half of the participants (56.9%) were married and about 41.5% had an undergraduate or postgraduate degree. A majority of the participants were Kenyan (97.5%) and about 69.0% were employed or self-employed. About 71.0% reported having no medical conditions whereas 8.7% reported having hypertension, 7.8% having diabetes, and 2.7% having high cholesterol. About 12.2% of the respondents reported that they had tested positive for COVID-19. However, about 43.7% reported that they might have been exposed to or infected with COVID-19 without testing. More than half of our study population reported fear of becoming infected (63.6%), fear of death from the infection (63.1%), and fear of a family member becoming infected (61.1%). From the responses, 3.8% mentioned all three worries (food insecurity/job related worries/financial related worries), whereas 26.9% mentioned any two worries (food insecurity/job related worries/financial related worries. Figure 1 details what our study population most worried about during the COVID-19 pandemic. Detailed demographics are presented in Table 1.

### 3.2. Attitudes and Perspectives toward COVID-19 Vaccines

In this study, 81.0% of the participants strongly agreed/agreed that it was important to get a vaccine to protect other people from COVID-19. Furthermore, about 61.6% of the participants agreed that pharmaceutical companies will be able to develop safe and effective COVID-19 vaccines and only 30.0% believed COVID-19 vaccines made in Europe or America were safer than those made in other countries. A majority of the participants (79.9%) reported they would take a vaccine to protect against COVID-19 yet 40.5% reported being hesitant to take the vaccine due to side effects from the vaccine. Recommending family and friends and getting children vaccinated against COVID-19 vaccine was reported at 77.6% and 65.4%, respectively.

More than half of the participants (65.1%) agreed that the COVID-19 vaccine was rapidly developed and approved and about 25.5% of the participants agreed the vaccines were faulty or fake. More than one-third of the participants (37.6%) agreed the vaccine might cause medical complications in the future and 26.8% stated they felt the vaccine was being promoted for commercial gain. Table 2 depicts the attitudes and perceptions towards the COVID-19 vaccine.

### 3.3. Vaccination Status Differences

Approximately 68.8% of the participants reported being vaccinated with at least one dose. From those that had received the vaccine, 72.0% received two doses, 25.7% received one dose, and 2.3% had received the additional booster dose. More than half (61.9%) reported getting the AstraZeneca vaccine (first vaccine available in Kenya). Variables that were investigated as potential associated variables of receiving COVID-19 vaccines were: age, gender, marital status, education, employment, stating that vaccines are safe, COVID-19 exposure, and questions regarding attitudes and perceptions regarding COVID-19 vaccines. Most of the variables were associated with receiving a vaccine. Older age participants were more likely to receive a vaccine and in addition, unemployed participants and those with no or only school education were less likely to have received the COVID-19 vaccine as compared to employed participants and those with undergraduate or postgraduate education. Furthermore, participants who believed that general vaccines are safe, in the importance of getting a vaccination against COVID-19, and that pharmaceutical companies develop safe and effective vaccines were more likely to have received a vaccine. In contrast, participants who believed COVID-19 vaccines may be faulty or fake, might cause some medical complications in the future, and that vaccines are being promoted for commercial gain were less likely to have received the vaccine. Details of the results from the logistic regression are presented in Table 3.

### 3.4. Institution Differences

We looked at differences among sociodemographic characteristics, COVID-19 related questions, and attitudes and perspectives towards COVID-19 vaccines. The institutional comparison was divided into “Private” (Aga Khan University Hospital, Avenue Hospital, Mediheal Hospital, and Penda Health), “Faith-Based” (PCEA Chogoria Hospital) and “Government” (Coast General Teaching and Referral Hospital). The distribution of participants from the three groups was 55.1% from private, 19.5% from faith-based, and 25.3% from government facilities. Most of the sociodemographic characteristics were associated with the type of institution. From the private institutions, 59.0% were married, 51.0% had an undergraduate or postgraduate education, and 81.2% reported being employed, which was higher compared to those at the faith-based and government institutions. Only about half of the participants from the government and faith-based facilities, 52.1% and 54.5%, respectively, were vaccinated, whereas 81.5% from the private facilities reported being vaccinated (*p* < 0.001). More participants from private facilities felt that general vaccines are safe compared to those at the faith-based and government facilities, which was statistically significant (*p* < 0.001). From the faith-based facility, 42.5% agreed on COVID-19 vaccines being faulty or fake, whereas of those at the government facility and the private institutions, 29.3% and 17.7%, respectively, of respondents agreed that the COVID-19 vaccine was faulty or fake (*p* < 0.001). About one-third of the participants (35.7%) from the government facility agreed that the COVID-19 vaccine might cause some medical complications in the future whereas about 60.6% from the faith-based facility reported the same. Details of the univariate analysis associations are presented in Table 4.

## 4. Discussion

Vaccine decision-making remains a complex matter defined by social, political, geographical, and cultural factors. It remains a major obstacle in the optimal administration of vaccines to populations in LMICs. Vaccine hesitancy has been inadequately studied in Africa and especially in Kenya, hampering efforts to mitigate these challenges. We chose to further explore the vaccine hesitancy among patients visiting six healthcare facilities in Kenya to add to the scarce data on vaccine hesitancy from sub-Saharan Africa.

Our study showed about 40.5% of the respondents were hesitant to take the vaccine, 81% felt that it was important to be vaccinated against COVID-19, and 77% stated that they would recommend the vaccine to family members and friends. Studies in SSA vary with some studies showing similar numbers while others in Cameroon and the DRC reporting much higher rates of vaccine hesitancy [14,30,31,32]. Media, trust in pharmaceutical companies, lack of information about the vaccines, as well as cost, play a key role in vaccine hesitancy in countries with a higher vaccine hesitancy rate. Approximately 63% of our study population received both dose of primarily the AstraZeneca vaccine. This was the first vaccine made available in Kenya in early 2021 through COVAX—a partnership between CEPI, Gavi, UNICEF, and the WHO [33]. Other approved vaccines reached the country later in 2021.

The depth of vaccine acceptance in our population is reflected in that 65% were agreeable to vaccinate their children against COVID-19. Carcelen and colleagues also found a high acceptability of COVID-19 vaccinations for children brought in by parents for other routine vaccines in Zambia [34]. Concerns about the vaccine ranged from a relative preference for vaccines developed in the West to those based on conspiracy theories; 30% of our study population felt that vaccines developed in the West were of better quality, while 65% felt that the vaccine was rapidly developed and approved for use. In comparison, 28.5% in a study done in Egypt also reported they preferred vaccines from the United States [35]. This appears to reflect mistrust in the local healthcare systems as well as misinformation about research and safety of vaccines used specifically in Africa [36,37,38].

Approximately 25% of the study population felt that the COVID-19 vaccine was either faulty or fake. Similar findings from Zambia and the UK have been reported by other authors raising concerns about vaccine safety and effectiveness [34,39,40]. Conspiracy theories, such as Africans being experimental subjects for Western vaccines, can greatly affect the public’s views on vaccine safety and effectiveness [8,10,30,41]. Almost 47% of the population of Kenya believe that people in Africa are being used as test subjects for vaccine trials, whereas 88% had seen articles stating that the COVID-19 virus had been spread by China, and 71% had forwarded or shared this information with families and friends [42].

The COVID-19 vaccines are being promoted for commercial gain, 27% of our study population felt, similar to findings of other studies. Prior studies have documented a similar mistrust in the intentions of the pharmaceutical industries in Africa, and that they are prioritizing financial gains over public welfare [30]. A majority of our study population also felt that the vaccines should be provided at no cost to the public even though currently the country is providing them for free. It was reported by other studies as well that, specifically in Africa, the cost of the vaccine may be associated with non-acceptance of the vaccine [15,30,43].

It was clear from our study that our respondents’ risk perception was related to their attitude about that vaccine. Similar to other studies, our study showed that higher levels of education and increasing age were associated with getting a COVID-19 vaccine [44,45,46,47]. This is contrary to what the Africa CDC found in its study reporting that people with primary level education and less were more likely to show confidence in the importance and safety of the vaccine, but they also reported that older people were more likely to get the COVID-19 vaccine; 20% of Kenyans aged 18–24 were not willing to take the vaccine versus 7% over the age of 55 [42]. The difference could be partially attributed to the sample size and location of study and certainly deserves further analysis. Participants in our study who believed the COVID-19 vaccine maybe faulty or fake were less likely to have received the vaccine. Our findings were substantiated by the CDC Africa report showing similar findings [42].

Our study, to the best of our knowledge, is the first study to compare vaccine hesitancy among the different types of healthcare institutions in Kenya. Further analysis looked specifically at attitudes and perceptions of the COVID-19 vaccine comparing government facilities to private and faith-based health institutions.

A significant number of participants at the faith-based hospital (42%) felt that the COVID-19 vaccine was fake or faulty. This was in comparison to 29% and 17% at the government and private hospitals, respectively. Similarly, a majority of the participants (82%) at the faith-based hospital felt that the COVID-19 vaccine was rapidly developed and approved compared to 53% and 64% at the government and private hospitals, respectively. It is unclear why the participants at the faith-based hospital felt this way and certainly this question needs further exploration. We also found that healthcare providers followed by scientific articles were the most trusted source of information about COVID-19 vaccines among those in private institutions, whereas in government and faith-based facilities, healthcare providers followed by media sources were most trusted sources for information on the COVID-19 vaccine. This is in contrast to what CDC Africa reported: that social media and newspapers were the most popular source of information about the vaccine in Kenya [42]. Again, this difference could be explained by the varied geographical sample population. Similar to other studies and probably true of our study as well is that misinformation on social media could have accounted for lower rates of trust in the vaccine in the government and faith-based facilities [11,48].

Approximately 53% of the participants in the government hospital had no formal or high school education. This was in comparison to the 51% of the participants in the private institutions who had an undergraduate or postgraduate degree and 43% of the participants in the faith-based hospital who had a diploma. Level of education has been shown to be a positive predictor of vaccine acceptance in the United States and the Middle East, but not necessarily in sub-Saharan Africa [11,14].

Vaccine uptake at the government hospital in Mombasa and the faith-based facilities was around 52% and 55%, respectively, compared to 82% at the private facilities (all located in Nairobi). This geographically aligns with reports from the Government of Kenya, stating that the vaccination rate as of December 2021 in Nairobi remained the highest, approximately 1.3 million, and was much lower in Mombasa at around 160,000 people around the same time [49]. We postulate that difference in education levels among participants at the different sites as well as the primary source of information about the vaccine played a significant role in vaccine hesitancy within the government and faith-based hospitals.

In this study, approximately 48% of the population seeking care at the government hospital and 46% of the population seeking care at faith-based hospital reported not receiving the vaccine compared to only 19% of the population at private hospitals in Kenya. These findings could be explained by the education level of the participants at the different sites as well as the primary source of information about the vaccine. In addition, participants from the government hospital and faith-based hospital in our study were more often than not from low income households, with higher rates of unemployment, compared to participants from the private institutions. Our study also showed that 11% of the participants within the government hospital and 7% within the faith-based hospital did not feel that it was important to get vaccinated to protect others. This is in comparison to 3% within the private hospitals, and could be potentially explained by the level of education among the different participants as well as the key sources of information about the vaccine. Concerns about the side effects of the vaccine were reported by 71% of participants from the faith-based hospital compared to 40% and 29% at the government and private hospitals, respectively. We are not sure if religious backgrounds and affiliations had an impact on vaccine hesitancy in our study. However, some authors in the West have reported no association between religion and vaccine hesitancy and this question needs further exploration in SSA [50].

Our study has several limitations. Firstly, our study population was recruited at outpatient and inpatient healthcare facilities. Patients actively attending a healthcare facility may be more likely to perceive a need to get the vaccine due to medical illness, and may be more likely to access health services of any type. Hence our study population might not be truly reflective of the general population in Kenya, especially the Kenyans living in rural areas. Secondly, we conducted surveys in four private hospitals, one government and one faith-based hospital, hence making generalization of our results difficult, especially concerning government and faith-based facilities. Thirdly, our survey was conducted in English and it was more difficult for non-English speakers to participate. Lastly, even though anonymous, the surveys were conducted in a hospital setting, so some patients might have felt uncomfortable disclosing their true vaccination status for fear of being stereotyped.

In conclusion, vaccine hesitancy in Kenya, even though much lower than reported in other countries, remains a dynamic and challenging problem in LMIC. Lack of trust in the healthcare system, and misinformation about the vaccine and side effects of the vaccine, remain barriers to effectively vaccinating the public. Although theoretically available, access to vaccines especially in rural areas still continues to pose a challenge. Mitigating strategies specific to Africa need to be developed to help address vaccine hesitancy in this part of the world.

## Figures and Tables

**Table 1:** Demographic details of the study participants (*n* = 3996).

Variable		*n*	%
Study Site	Aga Khan University, Nairobi	925	23.1%
	Penda Health	345	8.6%
	Avenue Hospital	517	12.9%
	Mediheal Hospital	417	10.4%
	Coast General Hospital	1012	25.3%
	PCEA Hospital	780	19.5%
Age (years) (*n* = 3624)	Median [IQR]	33.0 [26.5, 43.0]	
	18–25 years	767	21.2%
	26–35 years	1322	36.5%
	>35 years	1535	42.4%
Gender (*n* = 3979)	Male	1759	44.2%
	Female	2220	55.8%
Marital Status (*n* = 3947)	Single	1509	38.2%
	Married	2245	56.9%
	Other	193	4.9%
Education (*n* = 3947)	None/School Education	1051	26.6%
	Diploma	1258	31.9%
	Undergraduate/Postgraduate	1638	41.5%
Employment (*n* = 3953)	Employed/Self Employed	2726	69.0%
	Unemployed	1117	28.3%
	Retired	110	2.8%
Nationality (*n* = 3975)	Kenyan	3874	97.5%
	Non-Kenyan	101	2.5%
Race (*n* = 3959)	African	3843	97.1%
	Other	116	2.9%
	Asian	79	2.0%
	Other	12	0.3%
Medical Conditions	Diabetes	312	7.8%
	Hypertension	346	8.7%
	Heart Disease	81	2.0%
	High Cholesterol	107	2.7%
	Stroke	20	0.5%
	HIV	171	4.3%
	Cancer	41	1.0%
	Other	267	6.7%
	None	2837	71.0%
Previously tested positive for COVID-19 (*n* = 3981)	Myself	487	12.2%
	Family Member	905	22.7%
	Friend	878	22.1%
	Colleague	790	19.8%
	My Neighbor	504	12.7%
	No One	1427	35.8%
Exposed to or infected with COVID-19 (without testing) (*n* = 3932)	Yes	1720	43.7%
	No	2212	56.3%

**Table 2:** Attitudes and perceptions towards COVID-19 vaccine.

	Strongly Agree/Agree	Neutral	Strongly Disagree/Disagree
It is important to get a vaccine to protect people from COVID-19. (*n* = 3966)	3212 (81.0%)	513 (12.9%)	241 (6.1%)
Pharmaceutical companies are going to develop safe and effective COVID-19 vaccines? (*n* = 3891)	2395 (61.6%)	1111 (28.6%)	385 (9.9%)
COVID-19 vaccines made in Europe or America are safer than those made in other countries. (*n* = 3853)	1155 (30.0%)	1318 (34.2%)	1380 (35.8%)
I would take a vaccine to protect against COVID-19. (*n* = 3953)	3159 (79.9%)	425 (10.8%)	369 (9.3%)
I am hesitant to take the vaccine due to side effects from the vaccine. (*n* = 3905)	1580 (40.5%)	581 (14.9%)	1744 (44.7%)
I will recommend my family and friends to get vaccinated against the COVID-19 vaccine? (*n* = 3954)	3070 (77.6%)	521 (13.2%)	363 (9.2%)
I will get my children vaccinated against COVID-19? (*n* = 3704)	2421 (65.4%)	658 (17.8%)	625 (16.9%)
COVID-19 vaccine maybe faulty or fake. (*n* = 3942)	1005 (25.5%)	1304 (32.6%)	1633 (41.4%)
COVID-19 vaccine was rapidly developed and approved. (*n* = 3917)	2550 (65.1%)	912 (23.3%)	455 (11.6%)
COVID-19 vaccine might cause some medical complications in the future. (*n* = 3921)	1474 (37.6%)	1551 (39.6%)	896 (22.9%)
COVID-19 vaccine is being promoted for commercial gain. (*n* = 3906)	1046 (26.8%)	1318 (33.7%)	1542 (39.5%)
The government should make the vaccine available for all citizens for free. (*n* = 3931)	3408 (86.7%)	342 (8.7%)	181 (4.6%)

**Table 3:** Association of factors among those vaccinated.

Factors		Odds Ratio	95% CI	*p* Value
Age (years)		1.029	1.023–1.036	<0.001
Gender (Ref: Female)	Male	1.044	0.912–1.195	0.53
Marital Status (Ref: Single)	Married	1.443	1.256–1.658	<0.001
	Others	2.721	1.852–3.998	<0.001
Education (Ref: Undergraduate/Postgraduate)	None-Primary	0.422	0.358–0.498	<0.001
	Diploma	0.833	0.706–0.983	0.030
Employment (Ref: Employed/Self-employed)	Unemployed	0.293	0.253–0.340	<0.001
	Retired	0.779	0.509–1.191	0.25
In general, vaccines are safe (Ref: Neutral)	Agree	2.994	2.507–3.576	<0.001
	Disagree	0.395	0.284–0.549	<0.001
Previously tested positive for COVID-19 (Ref: No)	Self	2.458	1.919–3.148	<0.001
	No One	0.415	0.361–0.476	<0.001
Infected with COVID-19 (without testing) (Ref: No)	Yes	2.223	1.927–2.564	<0.001
Important to get a vaccine to protect people from COVID-19 (Ref: Neutral).	Agree	8.051	6.560–9.789	<0.001
	Disagree	0.530	0.366–0.766	0.001
Pharmaceutical companies are going to develop safe and effective COVID-19 vaccines (Ref: Neutral).	Agree	2.295	1.968–2.676	<0.001
	Disagree	0.401	0.316–0.509	<0.001
COVID-19 vaccines made in Europe or America are safer than those made in other countries (Ref: Neutral).	Agree	1.118	0.940–1.329	0.21
	Disagree	0.827	0.704–0.971	0.021
I am hesitant to take the vaccine due to side effects from the vaccine (Ref: Neutral).	Agree	0.589	0.485–0.716	<0.001
	Disagree	3.680	2.962–4.574	<0.001
I will recommend my family and friends to get vaccinated (Ref: Neutral).	Agree	7.701	6.298–9.417	<0.001
	Disagree	0.449	0.327–0.616	<0.001
I will get my children vaccinated (Ref: Neutral).	Agree	3.223	2.681–3.874	<0.001
	Disagree	0.372	0.296–0.467	<0.001
COVID-19 vaccines may be faulty or fake (Ref: Neutral),	Agree	0.417	0.352–0.495	<0.001
	Disagree	2.266	1.906–2.694	<0.001
COVID-19 vaccine was rapidly developed and approved (Ref: Neutral),	Agree	1.276	1.086–1.499	0.003
	Disagree	0.885	0.700–1.118	0.30
COVID-19 vaccines might cause some medical complications in the future (Ref: Neutral)	Agree	0.341	0.292–0.399	<0.001
	Disagree	1.442	1.172–1.774	0.001
The COVID-19 vaccine is being promoted for commercial gain (Ref: Neutral),	Agree	0.490	0.414–0.579	<0.001
	Disagree	2.018	1.698–2.397	<0.001

**Table 4:** Differences among institutions.

		Institution						*p* Value
		Private (*n* = 2204)		Faith Based (*n* = 780)		Govt (*n* = 1012)		
Age (years) (*n* = 3624) (median [IQR])		34.0 [28.0, 45.0]		30.0 [23.0, 41.0]		33.0 [26.0, 41.0]		<0.001
Gender (*n* = 3979)	Male	991	45.2%	319	40.9%	449	44.5%	0.109
	Female	1200	54.8%	461	59.1%	559	55.5%	
Marital Status (*n* = 3947)	Single	796	36.7%	368	47.4%	345	34.4%	<0.001
	Married	1280	59.0%	388	50.0%	577	57.6%	
	Others	93	4.3%	20	2.6%	80	8.0%	
Education (*n* = 3947)	None/School Education	337	15.5%	187	24.0%	527	52.7%	<0.001
	Diploma	726	33.5%	334	42.9%	198	19.8%	
	Undergraduate/Postgraduate	1105	51.0%	258	33.1%	275	27.5%	
Employment (*n* = 3953)	Employed/Self Employed	1763	81.2%	341	43.8%	622	61.9%	<0.001
	Unemployed	332	15.3%	431	55.4%	354	35.2%	
	Retired	75	3.5%	6	0.8%	29	2.9%	
Nationality (*n* = 3975)	Kenyan	2098	95.9%	775	99.6%	1001	99.2%	<0.001
	Non-Kenyan	90	4.1%	3	0.4%	8	0.8%	
Race (*n* = 3959)	African	2088	95.7%	778	99.9%	977	97.9%	<0.001
	Others	94	4.3%	1	0.1%	21	2.1%	
Medical Conditions	None	1492	67.7%	578	74.1%	767	75.8%	<0.001
Medical Conditions	Diabetes	156	7.1%	79	10.1%	77	7.6%	0.023
	Hypertension	203	9.2%	75	9.6%	68	6.7%	0.038
	Heart Disease	28	1.3%	26	3.3%	27	2.7%	0.01
	High Cholesterol	66	3.0%	12	1.5%	29	2.9%	0.088
	Stroke	15	0.7%	3	0.4%	2	0.2%	0.173
	HIV	125	5.7%	31	4.0%	15	1.5%	<0.001
	Cancer	11	0.5%	12	1.5%	18	1.8%	0.001
In general, vaccines are safe (*n* = 3978).	Agree	1778	81.2%	641	82.5%	719	71.2%	<0.001
	Neutral	348	15.9%	92	11.8%	176	17.4%	
	Disagree	65	3.0%	44	5.7%	115	11.4%	
Previously tested positive for COVID-19.	Myself	332	15.1%	82	10.5%	73	7.2%	<0.001
	No One	626	28.4%	351	45.0%	450	44.5%	<0.001
Exposed to or infected with COVID-19 (without testing) (*n* = 3932).	Yes	1120	51.8%	375	48.6%	225	22.6%	<0.001
	No	1044	48.2%	397	51.4%	771	77.4%	
Most worried about during the COVID-19 pandemic.	Fear of becoming infected myself	1349	61.2%	501	64.2%	674	66.6%	0.010
	Fear of a family member becoming infected	1304	59.2%	503	64.5%	619	61.2%	0.031
	Death	1378	62.5%	572	73.3%	556	54.9%	<0.001
	Financial-related worries	957	43.4%	358	45.9%	588	58.1%	<0.001
	Job-related worries	656	29.8%	187	24.0%	574	56.7%	<0.001
	Food insecurity-related worries	233	10.6%	55	7.1%	218	21.5%	<0.001
	Unavailability of vaccines	387	17.6%	63	8.1%	177	17.5%	<0.001
	Being a plot or conspiracy	203	9.2%	65	8.3%	137	13.5%	<0.001
	Being forced to take a medication	134	6.1%	64	8.2%	90	8.9%	0.008
	Being forced to take a vaccine	257	11.7%	85	10.9%	137	13.5%	0.182
	Not worried about any issues	89	4.0%	60	7.7%	30	3.0%	<0.001
Received the COVID-19 vaccine?	Yes	1796	81.5%	425	54.5%	527	52.1%	<0.001
	No	408	18.5%	355	45.5%	485	47.9%	
How many doses of the vaccine did you receive?	One	385	21.4%	138	32.5%	183	34.7%	<0.001
	Two	1352	75.3%	287	67.5%	340	64.5%	
	Three	59	3.3%	0	0.0%	4	0.8%	
Do you think it is important to get a vaccine to protect people from COVID-19?	Agree	1879	85.9%	617	79.1%	716	71.7%	<0.001
	Neutral	231	10.6%	109	14.0%	173	17.3%	
	Disagree	77	3.5%	54	6.9%	110	11.0%	
Pharmaceutical companies are going to develop safe and effective COVID-19 vaccines?	Agree	1435	67.6%	407	52.4%	553	55.7%	<0.001
	Neutral	572	27.0%	295	38.0%	244	24.6%	
	Disagree	115	5.4%	75	9.7%	195	19.7%	
COVID-19 vaccines made in Europe or America are safer than those made in other countries?	Agree	550	26.3%	184	24.2%	421	41.9%	<0.001
	Neutral	857	41.0%	274	36.1%	187	18.6%	
	Disagree	681	32.6%	302	39.7%	397	39.5%	
Which of the following COVID-19 vaccines would you prefer to use in the future?	Some Vaccine	1855	86.6%	624	80.1%	598	59.8%	<0.001
	None	287	13.4%	155	19.9%	402	40.2%	
I would take a vaccine to protect against COVID-19.	Agree	1852	85.1%	595	76.3%	712	71.4%	<0.001
	Neutral	205	9.4%	90	11.5%	130	13.0%	
	Disagree	119	5.5%	95	12.2%	155	15.5%	
I am hesitant to take the vaccine due to side effects from the vaccine.	Agree	626	29.4%	555	71.2%	399	40.1%	<0.001
	Neutral	332	15.6%	81	10.4%	168	16.9%	
	Disagree	1172	55.0%	144	18.5%	428	43.0%	
I will recommend my family and friends to get vaccinated against the COVID-19 vaccine?	Agree	1821	83.5%	591	76.2%	658	66.1%	<0.001
	Neutral	263	12.1%	124	16.0%	134	13.5%	
	Disagree	98	4.5%	61	7.9%	204	20.5%	
I will get my children vaccinated against the COVID-19 vaccine?	Agree	1346	69.7%	486	62.4%	589	59.3%	<0.001
	Neutral	373	19.3%	161	20.7%	124	12.5%	
	Disagree	212	11.0%	132	16.9%	281	28.3%	
COVID-19 vaccine maybe faulty or fake.	Agree	383	17.7%	330	42.4%	292	29.3%	<0.001
	Neutral	696	32.1%	302	38.8%	306	30.7%	
	Disagree	1087	50.2%	146	18.8%	400	40.1%	
COVID-19 vaccine was rapidly developed and approved.	Agree	1389	64.7%	636	81.5%	525	53.1%	<0.001
	Neutral	533	24.8%	107	13.7%	272	27.5%	
	Disagree	226	10.5%	37	4.7%	192	19.4%	
COVID-19 vaccine might cause some medical complications in the future.	Agree	651	30.2%	472	60.6%	351	35.7%	<0.001
	Neutral	994	46.1%	223	28.6%	334	33.9%	
	Disagree	513	23.8%	84	10.8%	299	30.4%	
COVID-19 vaccine is being promoted for commercial gain.	Agree	450	21.0%	332	42.7%	264	26.8%	<0.001
	Neutral	703	32.8%	279	35.9%	336	34.1%	
	Disagree	991	46.2%	167	21.5%	384	39.0%	
The government should make the vaccine available for all citizens for free?	Agree	1931	89.5%	727	93.2%	750	75.5%	<0.001
	Neutral	173	8.0%	44	5.6%	125	12.6%	
	Disagree	54	2.5%	9	1.2%	118	11.9%	
Would you be willing to pay for a COVID-19 vaccine privately?	Yes	375	17.4%	97	12.5%	66	6.7%	<0.001
	No	1318	61.3%	594	76.4%	755	76.1%	
	Not sure	458	21.3%	86	11.1%	171	17.2%	
Who do you trust the most for information about vaccines?)	Media (TV, radio, newspaper, etc.)	240	10.9%	304	39.0%	244	24.1%	<0.001
	Internet	150	6.8%	128	16.4%	77	7.6%	<0.001
	Social media (Facebook, Twitter, etc.)	90	4.1%	100	12.8%	102	10.1%	<0.001
	Healthcare providers	1301	59.0%	349	44.7%	254	25.1%	<0.001
	Family members	94	4.3%	42	5.4%	75	7.4%	0.001
	Government	379	17.2%	81	10.4%	138	13.6%	<0.001
	Pharmaceutical company reports	237	10.8%	60	7.7%	21	2.1%	<0.001
	Scientific articles	445	20.2%	86	11.0%	73	7.2%	<0.001
	I do not trust any source	208	9.4%	60	7.7%	94	9.3%	0.330

## Data Availability

According to our institutional information governance regulations, the anonymized data can be requested from the corresponding author.

## Supplementary Materials

The following supporting information can be downloaded at: https://www.mdpi.com/article/10.3390/vaccines10050705/s1. Questionnaire Survey.
