# Supplementary material for: Perceptions and Knowledge towards COVID-19 Vaccine Hesitancy among a Subpopulation of Adults in Kenya: An English Survey at Six Healthcare Facilities
Source: Vaccines (Basel). 2022 Apr 29;10(5):705. doi: 10.3390/vaccines10050705 (PMC9147716; doi:10.3390/vaccines10050705)
Supplement: Supplementary file 1 [file vaccines-10-00705-s001.zip › vaccines-1664679-supplementary.pdf]

Supplementary Appendix

**Demographics**	
**Study Site**	
**Gender**	☐ Male ☐ Female
**Current Age (years)**	_ _ _ _ _ _ _ _
**Marital status**	☐ Single ☐ Married ☐ Others
**Education (Degree)**	☐ None ☐ School education ☐ Diploma<br>☐ Undergraduate ☐ Postgraduate
**Employment**	☐ Employed ☐ Unemployed ☐ Retired
**Nationality**	☐ Kenyan ☐ Non-Kenyan
**Race**	☐ African ☐ Asian<br>☐ Caucasian ☐ Others Specify -------------------
**Medical History**	
**What medical conditions do you currently have**	☐ None ☐ Diabetes<br>☐ HTN ☐ High Cholesterol<br>☐ Heart Disease ☐ HIV<br>☐ Stroke ☐ Others Specify: ----------------------
**Attitudes towards Vaccines and Immunization**	
**In general, vaccines are safe.**	☐ Strongly agree ☐ Agree ☐ Neutral<br>☐ Disagree ☐ Strongly disagree
**COVID-19 Pandemic**	
**Who of the following tested positive for COVID-19?**	☐ Myself<br>☐ A family member<br>☐ A friend<br>☐ Colleague<br>☐ A neighbor<br>☐ No one
**Do you think that you may have been exposed to or infected with COVID-19 (without testing)?**	☐ Yes ☐ No
**What are you most worried about during this COVID-19 pandemic? (Tick not more than 5)**	☐ Fear of becoming infected myself<br>☐ Fear of a family member becoming infected

	☐ Death<br>☐ Financial related worries<br>☐ Job-related worries<br>☐ Food insecurity related worries<br>☐ Unavailability of vaccines<br>☐ Being a plot or conspiracy<br>☐ Being forced to take a medication<br>☐ Being forced to take a vaccine<br>☐ I am not worried about any issues<br>☐ Other
**Have your received the COVID-19 Vaccine?**	☐ Yes ☐ No
**Which vaccine did you receive?**	☐ Pfizer ☐ AstraZeneca/Covishield ☐ Moderna<br>☐ Sputnik ☐ Johnson & Johnson ☐ Others -----------
**How many doses of the vaccine did you receive?**	☐ 1 ☐ 2 ☐ Other
**Perspectives Toward COVID-19 Vaccination**	
**Do you think it is important to get a vaccine to protect the people from COVID-19?**	☐ Strongly agree ☐ Agree ☐ Neutral<br>☐ Disagree ☐ Strongly disagree
**Pharmaceutical companies are going to develop safe and effective COVID-19 vaccines?**	☐ Strongly agree ☐ Agree ☐ Neutral<br>☐ Disagree ☐ Strongly disagree
**Do you believe COVID-19 vaccines made in Europe or America are safer than those made in other world countries?**	☐ Strongly agree ☐ Agree ☐ Neutral<br>☐ Disagree ☐ Strongly disagree
**Which of the following COVID-19 vaccine do you prefer to use in the future?**	☐ Pfizer ☐ AstraZeneca/Covishield ☐ Moderna<br>☐ Sputnik ☐ Johnson & Johnson ☐ Others -----------<br>☐ None
**I would take a vaccine to protect against COVID-19**	☐ Strongly agree ☐ Agree ☐ Neutral<br>☐ Disagree ☐ Strongly disagree
**I am hesitant to take the vaccine due to side effects from the vaccine.**	☐ Strongly agree ☐ Agree ☐ Neutral<br>☐ Disagree ☐ Strongly disagree
**What side effects worry you the most about taking the COVID-19 vaccine?**	
**I will recommend my family and friends to get vaccinated against the COVID-19 vaccine?**	☐ Strongly agree ☐ Agree ☐ Neutral<br>☐ Disagree ☐ Strongly disagree
**I will get my children vaccinated against the COVID-19 vaccine?**	☐ Strongly agree ☐ Agree ☐ Neutral<br>☐ Disagree ☐ Strongly disagree

**COVID-19 vaccine maybe faulty or fake**	☐ Strongly agree ☐ Agree ☐ Neutral<br>☐ Disagree ☐ Strongly disagree
**COVID-19 vaccine was rapidly developed and approved**	☐ Strongly agree ☐ Agree ☐ Neutral<br>☐ Disagree ☐ Strongly disagree
**COVID-19 vaccine might have some medical complications in the future.**	☐ Strongly agree ☐ Agree ☐ Neutral<br>☐ Disagree ☐ Strongly disagree
**COVID-19 vaccine is being promoted for commercial gain**	☐ Strongly agree ☐ Agree ☐ Neutral<br>☐ Disagree ☐ Strongly disagree
**The government should make the vaccine available for all citizens for free?**	☐ Strongly agree ☐ Agree ☐ Neutral<br>☐ Disagree ☐ Strongly disagree
**Would you be willing to pay for a COVID-19 vaccine privately?**	☐ Yes ☐ No ☐ Not sure
**Who do you trust the most for information about vaccines?**	☐ Media (TV, Radio, Newspaper)<br>☐ Internet<br>☐ Social media (Facebook, Twitter, WhatsApp etc.)<br>☐ Health care providers: Physicians, pharmacists, etc.<br>☐ Family-members<br>☐ Government (JFDA)<br>☐ The pharmaceutical company reports<br>☐ Scientific articles<br>☐ I do not trust any source
